# Impact of variant-level batch effects on identification of genetic risk factors in large sequencing studies

**DOI:** 10.1371/journal.pone.0249305

**Published:** 2021-04-16

**Authors:** Daniel P. Wickland, Yingxue Ren, Jason P. Sinnwell, Joseph S. Reddy, Cyril Pottier, Vivekananda Sarangi, Minerva M. Carrasquillo, Owen A. Ross, Steven G. Younkin, Nilüfer Ertekin-Taner, Rosa Rademakers, Matthew E. Hudson, Liudmila Sergeevna Mainzer, Joanna M. Biernacka, Yan W. Asmann

**Affiliations:** 1 Division of Computational Biology, Department of Quantitative Health Sciences, Mayo Clinic, Jacksonville, Florida, United States of America; 2 National Center for Supercomputing Applications, University of Illinois at Urbana-Champaign, Urbana, Illinois, United States of America; 3 Division of Computational Biology, Department of Quantitative Health Sciences, Mayo Clinic, Rochester, Minnesota, United States of America; 4 Department of Neuroscience, Mayo Clinic, Jacksonville, Florida, United States of America; 5 Department of Clinical Genomics, Mayo Clinic, Jacksonville, Florida, United States of America; 6 Department of Neurology, Mayo Clinic, Jacksonville, Florida, United States of America; 7 Carl R. Woese Institute for Genomic Biology, Carver Biotechnology Center and Department of Crop Sciences, University of Illinois at Urbana-Champaign, Urbana, Illinois, United States of America; CNR, ITALY

## Abstract

Genetic studies have shifted to sequencing-based rare variants discovery after decades of success in identifying common disease variants by Genome-Wide Association Studies using Single Nucleotide Polymorphism chips. Sequencing-based studies require large sample sizes for statistical power and therefore often inadvertently introduce batch effects because samples are typically collected, processed, and sequenced at multiple centers. Conventionally, batch effects are first detected and visualized using Principal Components Analysis and then controlled by including batch covariates in the disease association models. For sequencing-based genetic studies, because all variants included in the association analyses have passed sequencing-related quality control measures, this conventional approach treats every variant as equal and ignores the substantial differences still remaining in variant qualities and characteristics such as genotype quality scores, alternative allele fractions (fraction of reads supporting alternative allele at a variant position) and sequencing depths. In the Alzheimer’s Disease Sequencing Project (ADSP) exome dataset of 9,904 cases and controls, we discovered hidden variant-level differences between sample batches of three sequencing centers and two exome capture kits. Although sequencing centers were included as a covariate in our association models, we observed differences at the variant level in genotype quality and alternative allele fraction between samples processed by different exome capture kits that significantly impacted both the confidence of variant detection and the identification of disease-associated variants. Furthermore, we found that a subset of top disease-risk variants came exclusively from samples processed by one exome capture kit that was more effective at capturing the alternative alleles compared to the other kit. Our findings highlight the importance of additional variant-level quality control for large sequencing-based genetic studies. More importantly, we demonstrate that automatically filtering out variants with batch differences may lead to false negatives if the batch discordances come largely from quality differences and if the batch-specific variants have better quality.

## Introduction

Genetic studies have shifted from Single Nucleotide Polymorphism (SNP) chip-based genome-wide association study (GWAS) of common variants to exome and whole-genome sequencing-based associations of rare variants. The large samples required for statistical power in sequencing-based searches for rare disease-associated variants often inadvertently introduce batch effects and systematic biases. Batch effects refer to sources of variation arising not from the targeted biological differences between sample classes but from differences between experimental or technological groups of samples [1]. If not adequately addressed in the analysis, batch effects reduce statistical power and lead to both false-positive and false-negative associations. Practices in large sequencing studies that commonly introduce batch effects include dividing samples among multiple sequencing centers [2, 3], collecting or preparing samples under different protocols [4], and extracting exomes using different target capture kits [4, 5]. For example, the Alzheimer’s Disease Sequencing Project (ADSP) sequenced exomes of more than 10,000 cases and controls to identify genetic factors associated with Alzheimer’s disease (AD) [6]. Sequencing of ADSP samples took place at three centers. Center 1 prepared sequencing libraries using the Illumina Rapid Capture Exome kit, while Center 2 and Center 3 used the Roche NimbleGen VCRome v2.1 kit.

A standard approach to manage batch effects is to identify and visualize batch effects using Principal Components Analysis (PCA) of the variant genotypes, and then either to adjust association models using batch covariates or to exclude batch-associated variants from further analysis [7–11]. However, this approach, which was developed during the SNP chip GWAS era, may not be sufficient for large genetic studies by sequencing [12]. The qualities and characteristics of variants identified by sequencing vary substantially even when all variants included in the association analysis have passed quality control thresholds such as those defined by the Variant Quality Score Recalibration (VQSR) model from the Genome Analysis Toolkit (GATK) [13]. Some quality-related characteristics differ between samples at the single-variant level. For example, exome kit capture efficiency may differ between the reference and alternative alleles, and the template amplification and sequencing chemistry differ between variants located in GC-rich and AT-rich regions. These variations will lead to differences in depth of coverage (DP), genotype quality (GQ) and alternative allele fraction (AAF; fraction/percentage of the sequencing reads aligned to the variant positions that support the alternative allele), etc. Because the variants that pass VQSR are all treated equally for disease association analysis, the impact of differences in individual variant-level qualities and characteristics is often masked.

We studied the ADSP cohort of 9,904 exomes and found that conventional PCA of the genotypes did not reveal the magnitude of the batch differences between samples sequenced at three centers and processed by two exome target capture kits. Furthermore, genetic association analyses that adjusted for center batches did not sufficiently remove hidden batch differences at the variant level. We found differences between capture kits in both variant GQ and AAF that significantly impacted the identification of AD-associated risk variants. Our findings highlight the importance of additional variant-level quality control to help researchers find truly meaningful genetic variants that are masked by batch effects.

## Results

### Description of dataset

The Sequence Read Archive (SRA) files containing the raw sequencing data of 10,993 AD cases and controls were downloaded from dbGaP (https://www.ncbi.nlm.nih.gov/gap/) and converted to FASTQ read files using the SRA Toolkit (https://www.ncbi.nlm.nih.gov/books/NBK158899). Access to this public dataset was approved by dbGaP and the Institutional Review Boards of Mayo Clinic and the University of Illinois. The Alzheimer’s cases satisfied the National Institute on Aging and the Alzheimer’s Association criteria [14] for definite, possible or probable Alzheimer’s disease. These cases included patients with and without *APOE* [15] risk alleles. The controls were at least 60 years old, showed no sign of dementia based on cognitive testing, and scored low on risk assessment [6]. Of the 10,993 samples, 9,904 passed sample-level quality control (QC) [16] based on the following criteria: variant call rate >95% per sample for SNPs and >90% for INDELs; sequencing depths >10x for at least 90% of the variants; *APOE* genotype matching between cohort meta-data and sequenced genotypic data; average transition/transversion ratio > 2.75 in the exonic regions; FREEMIX sample contamination estimate < 0.02 [17]; sex check (PLINK F estimate > 0.7 for males and < 0.3 for females); no first-, second- or third-degree relatedness as defined by the KING-robust algorithm [18]; and removal of duplicate samples. Of the 9,904 samples passing sample-level QC, Center 1 contributed 4,427 samples; Center 2 contributed 3,260; and Center 3 contributed 2,217. No samples were sequenced by multiple centers. Of the 1,584,609 variants detected in 9,904 ADSP exomes, 166,947 variants passed VQSR and the additional filtering steps detailed in the methods section.

### Population substructure explains sample clusters in PCA

**Fig 1:**
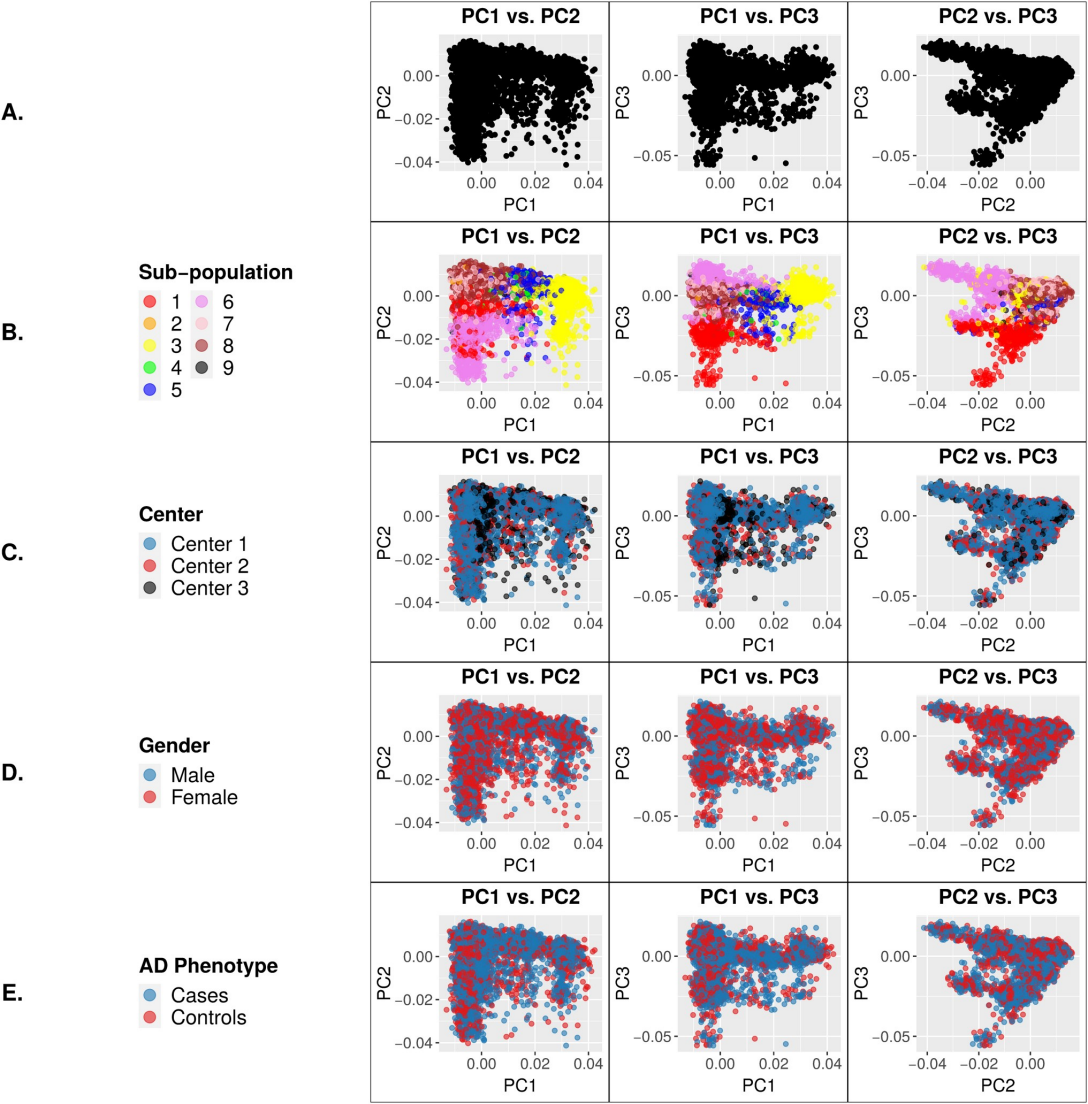
Principal Component (PC) eigenvector plots using genotypes of a pruned set of 16,187 high-quality common variants for 9,904 ADSP individuals. Each data point represents a single individual. Clustering of samples for a particular variable signifies genotypic similarity between individuals for the trait represented by that color. (A) PCs of the genotypes. (B) PCs color coded based on sub-population. (C) PCs color coded based on center. (D) PCs color coded based on gender. (E) PCs color coded based on AD phenotype. As expected, clustering is apparent only by sub-population.

The 9,904 ADSP subjects that passed sample-level QC were homogeneous based on 99.8% reporting European ancestry [16, 19] and on comparison to 1000 Genomes reference samples (S1 Fig). However, PCA of the ADSP genotypes using a pruned set of high-quality common variants (see Methods section) identified multiple sample clusters (Fig 1A) despite the homogeneous European ancestry of the study subjects. To determine whether the observed sample clusters represented population substructure or batch effects, we performed a *de novo* estimate using Admixture [20] that identified 9 sub-populations. As shown in Fig 1B, these 9 sub-populations overlapped with and accounted for the sample clusters identified in PCA. The sample batches of three different sequencing centers were not clearly visible by PCA, nor were gender or case/control status (Fig 1C–1E). Therefore, the sample clusters visualized by PCA can be explained largely by population substructure alone, at first glance. To control for this substructure, eigenvectors from the first four principal components were included as covariates in the association analysis. All 9,904 subjects were included in both the PCA and the association analysis.

### Association analysis

Of the 1,584,609 variants detected in 9,904 exomes, 166,947 variants passed the filtering criteria for association analysis detailed in the methods section (S2 Fig). Two association models were used. Model 1 adjusted for sequencing center and the first four principal components (PCs) from PCA underlying population substructure. Model 2 adjusted for sequencing center, the first 4 PCs, sex and *APOE* genotype. Neither model included age as a covariate because age confounds with disease status in this dataset by design. ADSP controls were deliberately selected to be younger than cases to favor the detection of disease-causal variants that are absent from older but cognitively normal individuals, and therefore previous ADSP association analyses have identified no significant variants under models adjusting for age [19], a finding that we replicated.

Combined, the association models identified 52 SNPs associated with AD with exome-wide significance, including 8 SNPs reported by the ADSP consortium [19] and 7 SNPs in the known AD genes *APOE* and *TOMM40* (S3 Fig). In this paper, we focus on 1) the 29 “novel” SNPs that remained significant after adjustment for *APOE* and sex and that have not previously been linked to AD, as well as on 2) the 7 SNPs in *APOE* and *TOMM40* as positive controls (Table 1).

**Table 1:** Seven SNPs in *APOE* and *TOMM40* (indicated by * of the SNP IDs) and 29 novel SNPs reaching exome-wide significance (*p* < 3.0 x 10^−7^, Bonferroni-corrected cutoff of *p* < 0.05 / # tests): Population minor allele frequency (MAF) in cases and controls, MAF in controls processed by Illumina or NimbleGen exome capture kit, and MAF in Non-Finish European (NFE) cohort of the ExAC database (http://exac.broadinstitute.org/).

Chr	Position	Ref	Alt	p-value (Model 1)	p-value (Model 2)	Gene	SNP ID	ADSP Cases MAF	ADSP Controls MAF	ADSP Controls MAF, Illumina	ADSP Controls MAF, NimbleGen	ExAC AAF, NFE
19	45411941	T	C	2.4E-185	N/A	APOE	rs429358*	0.2293	0.0701	0.074	0.067	0.1504
19	45396144	C	T	4.7E-103	0.7097	TOMM40	rs11556505*	0.2023	0.0879	0.085	0.090	0.0875
19	45395714	T	C	2.4E-75	0.1439	TOMM40	rs157581*	0.2766	0.1629	0.165	0.162	0.2326
19	45412079	C	T	1.8E-48	N/A	APOE	rs7412*	0.042	0.0988	0.116	0.087	0.0813
15	75913319	T	G	4.3E-45	2.7E-35	SNUPN	rs1004285543	0.0825	0.0255	0.063	0.001	NA
17	25973604	A	C	2.6E-45	1.4E-34	LGALS9	rs761436847	0.0823	0.0256	0.056	0.006	0.0906
6	36979483	T	G	1.7E-39	1.6E-29	FGD2	rs769719224	0.0998	0.0404	0.072	0.019	0.0294
14	99976645	A	C	4.1E-36	7.5E-28	CCNK	rs745936510	0.0899	0.0348	0.068	0.013	0.0713
13	114188430	C	T	4.7E-38	2.2E-27	TMCO3	rs77834374	0.1068	0.0459	0.080	0.024	0.1336
14	99976639	G	C	9.4E-36	3.7E-26	CCNK	rs778243462	0.0932	0.0372	0.071	0.015	0.0808
17	25973598	A	C	4.3E-25	1.1E-21	LGALS9	rs760143837	0.0472	0.014	0.033	0.002	0.0243
14	77706020	A	C	9.7E-28	6.3E-21	TMEM63C	rs774212969	0.0577	0.019	0.036	0.008	0.009
19	45397229	G	A	9.6E-21	0.3137	TOMM40	rs1160983*	0.0173	0.0416	0.045	0.039	0.0718
11	117280516	A	C	7.9E-29	2.5E-20	CEP164	rs756182128	0.0748	0.0296	0.050	0.016	0.081
3	42739737	T	G	5.7E-25	9.7E-20	HHATL	rs763168412	0.0539	0.0182	0.034	0.008	0.0919
3	48451952	A	C	3.2E-24	2.7E-19	PLXNB1	rs770786389	0.0562	0.02	0.037	0.009	0.0255
19	45397307	C	T	1.5E-18	0.928	TOMM40	rs112849259*	0.0308	0.0107	0.005	0.014	0.0011
12	56622883	A	C	4.3E-24	1.2E-17	NABP2	rs757798976	0.0714	0.0301	0.054	0.014	0.0476
2	85662149	A	C	4.0E-21	4.3E-16	SH2D6	rs748669078	0.068	0.0309	0.044	0.022	0.0026
19	10946797	G	C	6.4E-21	2.5E-15	TMED1	rs767166604	0.0421	0.0128	0.029	0.002	0.0007
14	105932775	G	C	5.5E-21	3.1E-15	MTA1	rs782227993	0.0627	0.0259	0.047	0.012	0.0208
6	29429950	A	C	1.1E-19	3.6E-15	OR2H1	rs746691570	0.0402	0.0132	0.022	0.007	0.0207
11	117280522	A	C	6.5E-21	3.3E-14	CEP164	rs758240656	0.0529	0.0198	0.037	0.009	0.0768
2	85662154	A	C	4.9E-18	4.0E-14	SH2D6	rs760146451	0.0617	0.0288	0.040	0.021	0.0018
13	88330245	A	C	3.1E-17	1.1E-13	SLITRK5	rs773717935	0.0277	0.0065	0.014	0.002	3.1E-05
19	45409167	C	G	9.7E-13	0.3854	APOE	rs440446*	0.3332	0.3817	0.361	0.395	0.4346
19	10946802	T	C	1.2E-16	3.1E-12	TMED1	rs776909029	0.0366	0.0117	0.028	0.001	0.0009
9	34564740	A	C	6.5E-16	3.6E-12	CNTFR	rs774039930	0.0516	0.0222	0.039	0.011	0.0008
3	108474687	T	G	1.4E-15	5.8E-12	RETNLB	rs199707443	0.0328	0.0107	0.025	0.001	0.0493
19	43025485	T	G	2.6E-15	7.7E-12	CEACAM1	rs763190977	0.0921	0.0523	0.107	0.016	0.0026
3	31659462	A	T	4.8E-17	9.2E-12	STT3B	rs74346226	0.0891	0.0514	0.076	0.035	0.131
12	109719316	T	G	9.1E-12	2.1E-10	FOXN4	rs760573591	0.0309	0.0115	0.025	0.003	1.5E-05
8	145112936	T	C	6.8E-14	5.0E-10	OPLAH	rs781948612	0.0331	0.0114	0.026	0.002	0.0364
19	42799299	T	C	5.7E-12	1.4E-09	CIC	rs745695673	0.019	0.0043	0.011	0.000	0
13	111164389	A	C	7.2E-12	1.6E-08	COL4A2	rs199702442	0.0517	0.0274	0.041	0.018	0.0285
22	30951295	T	G	2.0E-11	1.8E-08	GAL3ST1	rs762634521	0.0204	0.0056	0.013	0.001	0.028

Model 1 adjusted for sequencing center and the first four PCs underlying population substructure. Model 2 adjusted for sequencing center, the first 4 PCs, sex and *APOE* genotype.

### Batch effects between exome capture kits among AD-associated variants

**Fig 2:**
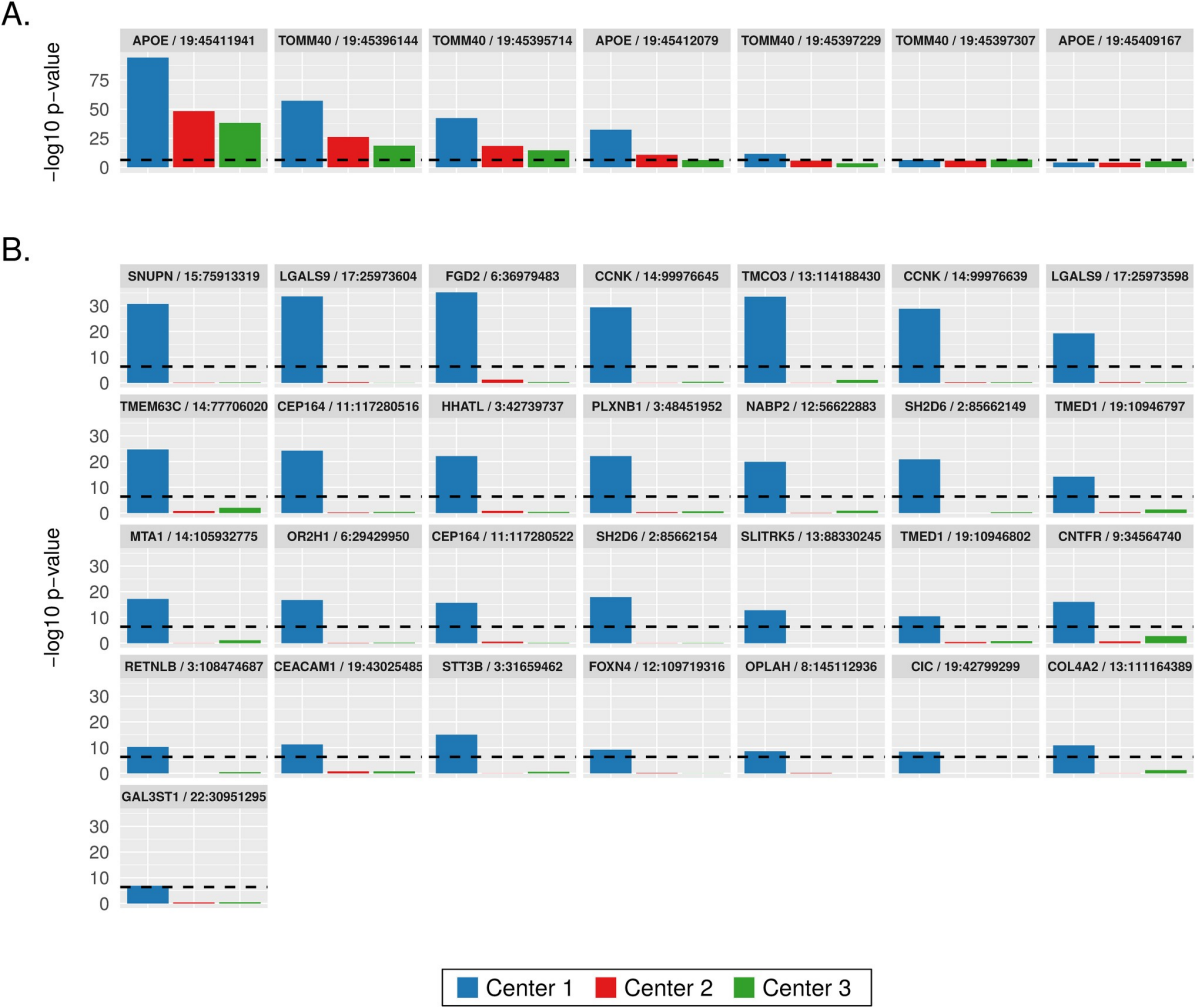
Sequencing center specific association p-values of SNPs that reached exome-wide significance (denoted by the dashed horizontal lines) in the full-dataset analysis. (A) Seven SNPs in *TOMM40* and *APOE*. (B) Twenty-nine novel SNPs.

Additional analyses of the 36 SNPs listed in Table 1 revealed that the significance of association came more from the individuals sequenced at Center 1 using the Illumina exome capture kit than from those sequenced at Center 2 and Center 3 using the NimbleGen exome capture kit. Association analyses of center-specific cohorts demonstrated that this center bias occurred in previously known AD SNPs in *APOE* and *TOMM40* (Fig 2A) as well as in the 29 novel SNPs (Fig 2B). The significance of AD association of the 29 novel SNPs came exclusively from the Center 1 cohort processed using the Illumina exome kit (Fig 2B), which may explain why the ADSP consortium did not report these SNPs.

**Fig 3:**
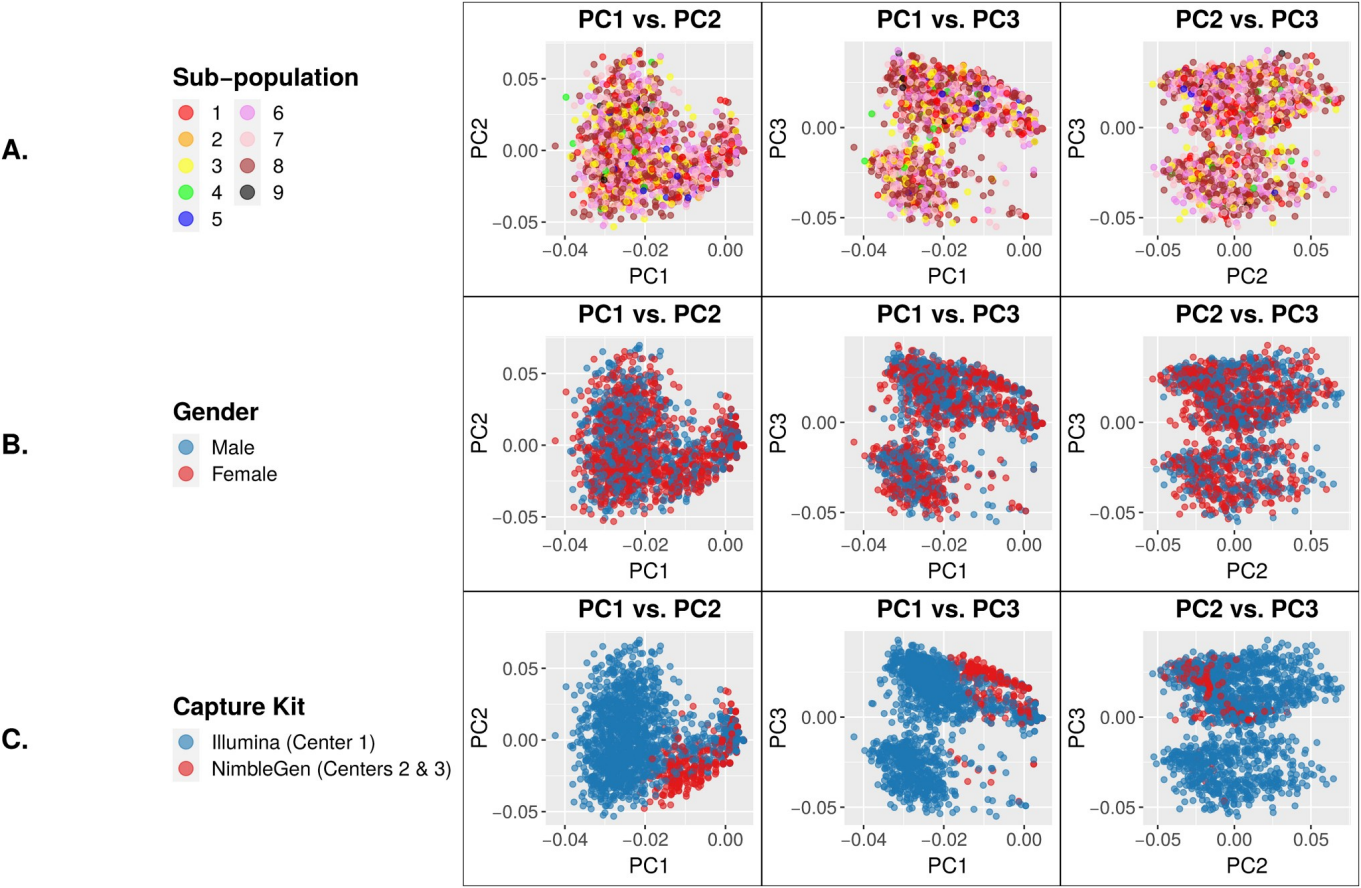
PC eigenvector plots of genotypes at 29 exome-wide significant SNPs. Each data point represents a single individual. Clustering of samples for a particular variable indicates genotypic similarity between individuals for the trait represented by that color. (A) PCs color coded based on sub-population. (B) PCs color coded based on gender. (C) PCs color coded based on capture kit. The NimbleGen-captured samples cluster tightly together, indicating their genotypic similarity that is distinct from the Illumina-captured samples.

We next studied these 29 novel SNPs using PCA. The SNP genotypes showed no differences between sub-populations or genders (Fig 3A and 3B). However, we observed a clear and significant difference between samples captured by the Illumina kit (Center 1) *vs*. those captured by the Roche NimbleGen kit (Center 2 and Center 3) (Fig 3C), consistent with the observation that these SNPs are only associated with AD in Center 1 samples as shown in Fig 2B. Since our association models included sequencing center as a covariate, the batch differences visualized in Fig 3C clearly were due to other factors, probably at the individual-variant level.

### Identification of variant-level differences in genotype quality and alternative allele fraction between two exome capture kits

**Fig 4:**
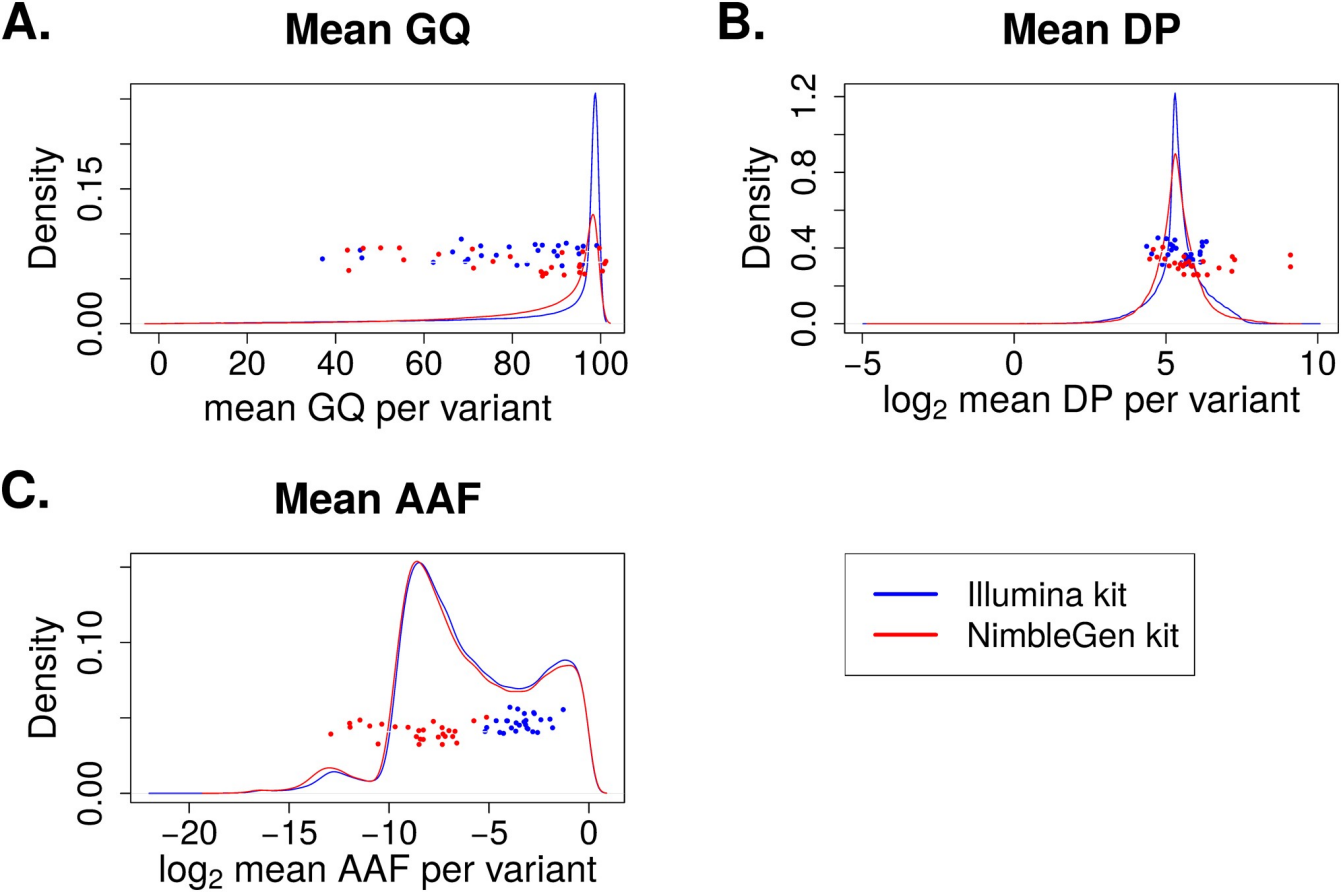
Density plots of variant quality parameters between two exome capture kits. Mean values were computed across all samples for each variant. The solid lines show the distributions of all 166,947 variants used in the association analyses, and the scattered dots represent the 29 novel SNPs. (A) Density plot for mean genotype quality (GQ). (B) Density plot for mean read depth (DP). (C) Density plot for mean alternative allele fraction (AAF).

**Fig 5:**
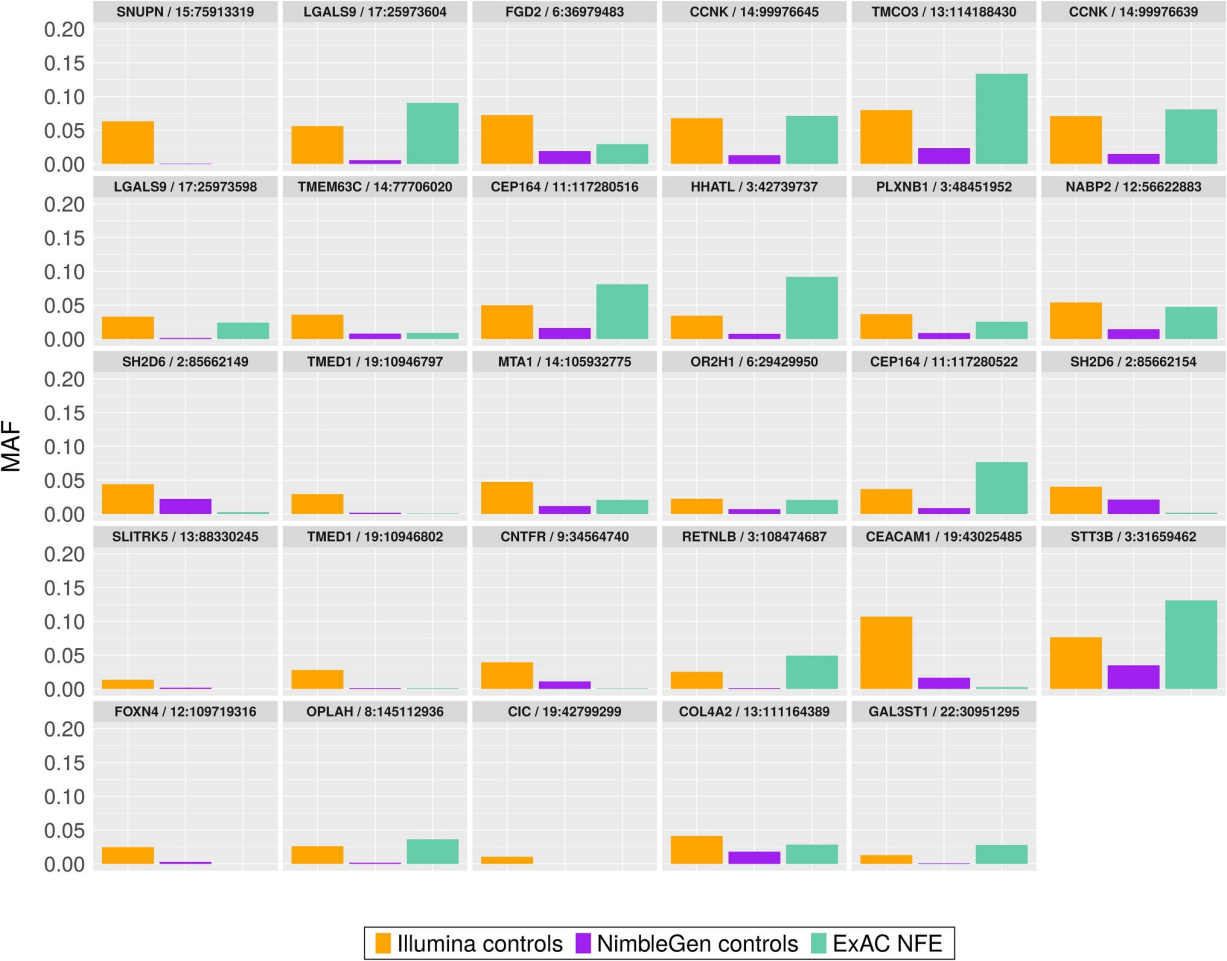
Minor Allele Frequency (MAF) of 29 exome-wide significant SNPs in AD control exomes processed by two capture kits and in the ExAC Non-Finnish European (NFE) population.

As the PCA plot of the 29 novel SNPs showed significant batch differences of genotypes between samples processed by two exome capture kits, we next examined variant-level factors that could explain why exclusively the Illumina kit-captured exomes yielded this set of highly significant novel SNPs. Although all analyzed SNPs passed VQSR quality thresholds, we speculated that there remained significant differences in qualities and characteristics at the individual-variant level between exome kits that might explain the greater significance of association in samples captured by one exome kit *vs*. the other. First, we compared several quality measures of the variants called in cohorts captured by the two kits. For the 166,947 SNPs used in the association analyses, we studied the distribution of the three most common variant quality parameters: GQ, DP and AAF (Fig 4). The distributions of mean GQ, DP, and AAF from 166,947 SNPs were very similar between capture kits. For the 29 SNPs of interest, the GQ (Fig 4A) and DP (Fig 4B) were also similar between capture kits. Interestingly, we observed a bi-modal distribution of AAF values from both capture kits, with the first mode closer to zero indicating approximately equal percentage of reads supporting reference and alternative alleles at the variant positions, and a second mode significantly deviated from zero and centered around log2 value of -6. This second mode represented variants with significantly lower percentage of reads supporting the alternative alleles than the reference alleles. More importantly, the AAFs of the 29 SNPs captured by the Illumina exome kit are mostly located close to the first mode and have a more balanced ratio of reference to alternative allele-supporting reads, while the AAFs of the 29 novel SNPs captured by the NimbleGen kit are located closer to the second mode. This observation implies that the NimbleGen kit lost most of the alternative alleles during exome capture, leading to the lack of variant calls in the NimbleGen cohort. Indeed, in the ADSP control group, the population minor allele frequencies (MAFs) of these 29 SNPs in the Illumina-captured cohort are generally higher compared to those in the NimbleGen-captured cohort, and the MAFs from the Illumina-captured cohort more closely resemble those in the Non-Finish European (NFE) population of the ExAC database (http://exac.broadinstitute.org/) (Table 1; Fig 5). This observation supports our hypothesis of more effective capture of the alternative alleles by the Illumina kit.

**Fig 6:**
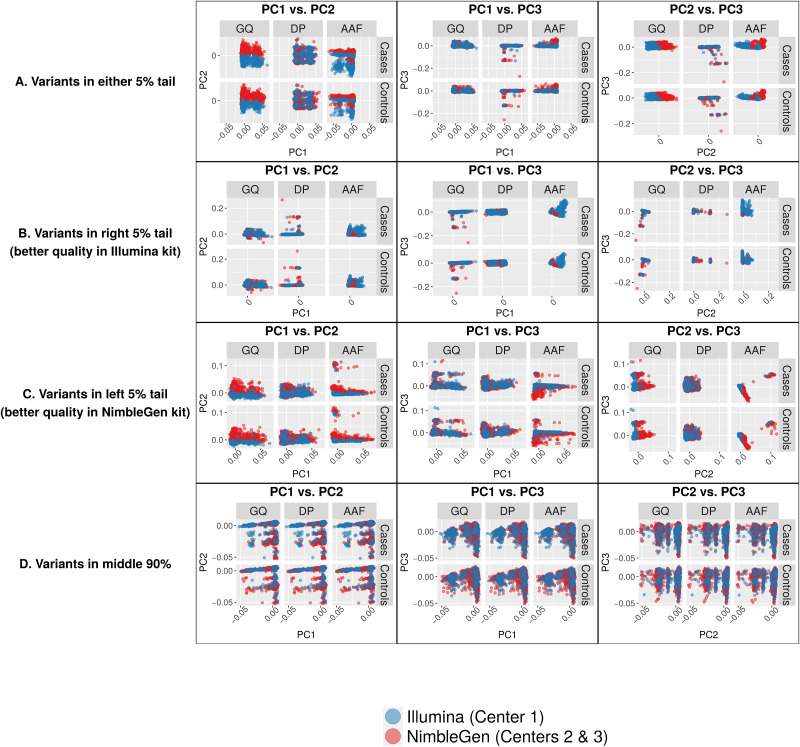
PC eigenvector plots of genotypes at variants lying in different sections of quality-metric ratio distributions. Each data point represents a single individual, color coded according to capture kit. (A) PCs of variants in either 5% tail. (B) PCs of variants in right 5% tail. (C) PCs of variants in left 5% tail. (D) Variants in middle 90% of distributions. Variants in the tails, in particular the left 5% tail (better quality in NimbleGen kit), show clear separation by capture kit in both cases and controls.

To further investigate variant quality differences between capture kits beyond the top 29 SNPs, we calculated distributions of log2 ratios of mean GQ, DP, and AAF between capture kits for all 166,947 variants (S4 Fig). We divided these variants into two groups: (1) the 10% of variants with the largest differences in GQ, DP, or AAF between two capture kits (5% at each of the two tails); and (2) the remaining 90% of variants. The PCA plots show that the 10% of variants with the biggest quality differences clearly contributed to the batch differences between the two exome capture kits (Fig 6A). Intriguingly, dividing these SNPs into those with better quality in Illumina-captured samples (right tail of the 5% variants; Fig 6B) and those with better quality in NimbleGen-captured samples (left tail of the 5% variants; Fig 6C) demonstrated that the batch differences came mostly from the latter. We observed no obvious batch contribution from the 90% of variants with similar GQ, DP, or AAF (Fig 6D).

## Discussion

We analyzed 9,904 exomes from ADSP to study the impact of batch effects on variant calling and association analysis. We focused on the known batches of three different sequencing centers and two exome capture kits. At first, no batch differences between sequencing centers or exome capture kits were visible from the PCA plots, and visually we were able to attribute all sample clusters in PCA to population substructure (Fig 1B). Sequencing center was included as a covariate in our association analyses; however, exome capture kit was not included in the association models because it confounds with sequencing center and therefore is not an independent variable. Besides the two models described in this paper, we analyzed the data using a third model which included age as a covariate. This third model identified no AD-associated variants because age confounds with disease status by design. Our results from Model 3 are similar to those reported by the ADSP consortium. Our association analyses identified 29 novel SNPs in addition to variants in previously known AD genes including *APOE* and *TOMM40* as well as variants reported by the ADSP consortium [19]. These 29 SNPs exemplified the impact of batch effects that were later attributed to the differences between the two exome capture kits. First, the significance of association with AD came exclusively from the samples processed by the Illumina exome kit at Center 1, while those processed by the NimbleGen kit at either Center 2 or Center 3 lacked significance (Fig 2B). Second, PCA of these 29 SNPs clearly showed separation of the samples according to capture kit (Fig 3C). The qualities of the variants between the two captured kits were similar overall at first glance but significantly different for a substantial number of variants (Figs 4 and 6). The fact that the majority of the variants had similar quality measures suggested that batch effects impacted individual variants rather than all variants.

Further analyses of the 29 SNPs of interest (Fig 4) pinpointed the difference in AAF (Alternative Allele Fraction; percent of reads supporting alternative allele) as a source of batch differences between the Illumina- and NimbleGen- processed cohorts. Although the distributions of the quality parameters GQ, DP, and AAF among all variants showed no differences between capture kits, the AAF of the 29 novel SNPs in particular were strikingly higher within the Illumina-processed cohort (Fig 4C), suggesting a batch effect that impacted individual variants rather than all variants. In addition, we observed a batch difference in GQ among the top 10% most different variants between the two capture kits (Fig 6A) and among the variants with better overall quality in the NimbleGen kit (Fig 6C). It is clear that the NimbleGen kit did not effectively capture the alternative alleles for these 29 SNPs, which implies that the variant calling of Illumina-captured was more reliable, and that the AD association of these 29 SNPs unique to the Illumina exome kit is likely real. We speculate that in previous publications the ADSP consortium applied an undisclosed filtering step to exclude variants with discordant significance between cohorts processed at different sequencing centers and/or by different exome capture kits, which would explain why these 29 SNPs have not been reported (e.g. [19]). Additional investigation of the genes connected to these 29 SNPs supports this speculation. Multiple genes underlying these 29 SNPs have previously been linked to neural function or pathology, including *Transmembrane P24 Trafficking Protein 1* (*TMED1*; rs767166604, association *p* = 7.22 x 10^−15^ for Illumina kit cohort; rs776909029, *p* = 3.25 x 10^−11^), *Plexin B1* (*PLXNB1*; rs770786389, *p* = 7.49 x 10^−23^), *Capicua Transcriptional Repressor* (*CIC*; rs745695673, *p* = 3.70 x 10^−9^), *Centrosomal Protein 164* (*CEP164*; rs756182128, *p* = 5.66 x 10^−25^; rs758240656, *p* = 1.94 x 10^−16^), and *Cyclin K* (*CCNK*; rs745936510, *p* = 3.98 x 10^−30^; rs778243462, *p* = 1.50 x 10^−29^). TMED1 is reported to interact with Amyloid Precursor Protein (*APP*) [21], whose cleavage into amyloid beta generates one of the key components of AD-associated pathological protein aggregation [22]. PLXNB1 influences amyloid beta load [23], and CIC is a transcriptional repressor whose inactivation promotes gliomagenesis, the formation of glial tumors in the brain [24]. CEP164 binds to TTBK2, a kinase that phosphorylates tau [25] and contributes to neurodegeneration in frontotemporal dementia [26]. Finally, copy-number mutations in the transcriptional regulator *CCNK* have been associated with neurodevelopmental abnormalities [27].

Because not all batch effects are created equal, no universal approach can manage all batch effects in every study. For large sequencing-based genetic studies, differences from sample storage/preparation methods, sequencing library construction protocols such as the choice of exome capture kits, and bioinformatics pipelines all result in batch differences that are potentially associated with data quality differences. The default approach today is to require concordant variant significances from all major batches and to ignore disease-associated variants if the signals were observed in one batch only. This practice will lead to potential false negatives. Instead, the key is to search for the source of batch-specific variant significance by checking variant-level quality parameters including, but not limited to, variant genotyping quality, mapping quality of the reads, sequencing depth, alternative allele fractions, base qualities, and the concordance of the MAF in the control cohort with public variant databases. As we demonstrated in our study, if the batch effects can be attributed to quality differences, the batch-associated variants should not be automatically discarded. We recommend that variants impacted by batch effects be included in validation studies using a different dataset or by wet lab functional assays.

## Conclusions

In summary, we discovered batch effects at the individual-variant level resulting from differences in GQ and AAF between different exome capture kits. In particular, we found that the signal of some variants most significantly associated with AD came exclusively from samples processed by one exome capture kit that was more effective at capturing the alternative alleles compared to the other kit. In general, a subset of variants with the biggest disparities in GQ and AAF values between the two exome capture kits contributed to the remaining batch effects. Our findings highlight the importance of additional variant-level quality control for large sequencing-based genetic studies. More importantly, we demonstrated that automatically filtering out variants with batch differences may lead to false negatives if the batch discordances come largely from quality differences and if the variants from one batch have better quality scores.

## Methods

### Ethics approval and consent to participate

All aspects of the study were approved the Institutional Review Boards of both Mayo Clinic and University of Illinois at Urbana-Champaign. This study was also approved by dbGAP. Written informed consent was obtained from all participants and surrogates by the ADSP consortium.

### Variant calling

The paired-end sequence reads were aligned to the human reference genome build 37 using Novoalign (http://www.novocraft.com) (default parameters), which was selected on the basis of its greater accuracy in read placement relative to other methods [28, 29] and its lack of prior application to this dataset for association testing (e.g. [19, 30]). The alignment files were then sorted by read position using Novosort (http://www.novocraft.com), realigned around small insertions and deletions (INDELs) using Picard (https://broadinstitute.github.io/picard/), and subjected to base recalibration using the Genome Analysis Toolkit (GATK) version 3.4 [31]. Variant calling followed GATK’s best practices guidelines for germline variants (https://gatk.broadinstitute.org/hc/en-us): per-sample variant calling on the realigned, recalibrated BAM files was performed using HaplotypeCaller, and multi-sample joint genotyping of all 9,904 samples was performed using GenotypeGVCFs. Variant calling was conducted only on the exome regions common between the two exome capture kits (Illumina Rapid Capture Exome kit and NimbleGen VCRome v2.1 kit). Variants were annotated by SnpEff [32] and ANNOVAR [33].

### Ancestry estimation

We implemented a series of filtering steps to select a pruned set of high-quality variants from the 1,584,609 variants detected across the cohort. Variants were excluded if they (1) failed VQSR; (2) lay within the highly variable HLA, LCT, 8p and 17q regions; (3) had MAF below 5% or deviation from Hardy-Weinberg Equilibrium below *p* < 1.0 E-4; or (4) had linkage disequilibrium *r*^*2*^ value above 0.2 within 0.1 megabase sliding windows. In total, 16,187 variants passed all filters, and 12,100 variants overlapped with variants detected in the 1000 Genomes samples of known ethnicities. The genotypes of the ADSP samples were combined with those of the 1000 Genomes samples at these 12,100 loci. PCs of the genotypes were then computed and plotted using the software plinkQC (https://meyer-lab-cshl.github.io/plinkQC/). The first four PCs of the ADSP genotypes had eigenvalues above 1 and were retained as covariates in the association analyses.

### Sub-population estimation

The software Admixture [20] was used to estimate sub-population number from the ADSP data *de novo*, using the 16,187 variants described above. Sub-population *k* values between 1 and 20 were tested using default settings. A value of 9 produced the lowest cross-validation standard error, indicating that 9 sub-populations best fit the data.

### Variant-level quality control for association analyses

Several steps were undertaken to minimize the number of false-positive variant calls prior to running the association models. The Variant Quality Score Recalibration (VQSR) step implemented in GATK uses machine learning algorithms to compute new, well-calibrated quality scores for each variant based on the annotations of a high-quality subset of the analyzed data. In accordance with GATK Best Practices for whole-exome data, the variables included in the VQSR model consisted of QD, MQ, MQRankSum, ReadPosRankSum, FS, SOR and InbreedingCoeff for SNPs; and QD, MQRankSum, ReadPOsRankSum, FS, SOR and InbreedingCoeff for INDELs [31]. A sensitivity threshold of 99.5 was used for SNPs and 99.0 for INDELs. Detected variants were excluded from association analysis if they failed VQSR, deviated significantly (*p* < 1.0 x 10^−6^) from Hardy-Weinberg equilibrium (HWE) in the control samples, or had an alternative allele call supported by fewer than 10 reads across the cohort (S2 Fig).

### Association tests, statistical model and variant filtering

After quality control, association testing using disease status (case or control) as the phenotype was performed on the variants under an additive logistic regression model implemented in Plink 1.9 [34]. PCs were calculated using Plink. The association tests were conducted on all 9,904 samples together for the full-cohort analysis, as well as on sets of samples stratified by sequencing center for the center-based association analyses. Variants were considered exome-wide statistically significant at the Bonferroni-corrected threshold of *p* < 0.05 / # tests, as in [19]. Only bi-allelic SNPs with a recalibrated variant quality score (VQSLOD) > 0 were retained for further analysis.

## Supporting information

S1 Fig Principal Component (PC) eigenvector plot of combined 1000 Genomes and ADSP genotypes. Each data point represents a single individual. 1000 Genomes reference individuals are color-coded by ancestry. ADSP samples are shown in black. The position of ADSP samples relative to the 1000 Genomes reference samples indicates their genotypic similarity, which reflects ancestry. Most ADSP samples cluster near European reference samples (e.g. Finland and Spain).
(DOCX)

S2 Fig Number of QCed variants used for association analysis, and their overlap among samples from three sequencing centers. These variants totaled 120,572 from the Center 1 samples; 108,390 from the Center 2 samples; and 98,542 from the Center 3 samples. Approximately 70% of variants were shared among samples from all three sequencing centers. The larger number of variants detected in Center 3 samples was likely due to the larger number of individuals sequenced by Center 1 compared to the other two centers.
(DOCX)

S3 Fig Log-transformed p-values of all 52 SNPs reaching exome-wide significance under any association model (largest p-value shown for each SNP). The red vertical line denotes exome-wide statistical significance (*p* < 3.0 x 10^−7^).
(DOCX)

S4 Fig Distributions of log-adjusted ratios of mean quality metrics for QCed variants shared between capture kits. Mean values were computed across all samples for each variant. Solid vertical lines demarcate the boundaries separating the 5% tails from the middle 90% of the distribution. The scattered dots represent the positions of the top 29 SNPs within the distributions. Almost all top SNPs lie far to the right of the mean AAF ratio distribution, indicating that these variants are highly discrepant between capture kits.
(DOCX)

## Abbreviations

AAF: Alternative Allele Fraction
ADSP: Alzheimer’s Disease Sequencing Project
DP: Sequencing Depth of Coverage
GATK: Genome Analysis Toolkit
GQ: Genotype Quality
GWAS: Genome-Wide Association Study
MAF: Minor Allele Frequency
PCA: Principal Components Analysis
QC: Quality Control
SNP: Single Nucleotide Polymorphism
VQSR: Variant Quality Score Recalibration
