# Supplementary figures and images for: Impact of variant-level batch effects on identification of genetic risk factors in large sequencing studies
Source: PLoS One. 2021 Apr 16;16(4):e0249305. doi: 10.1371/journal.pone.0249305 (PMC8051815; doi:10.1371/journal.pone.0249305)

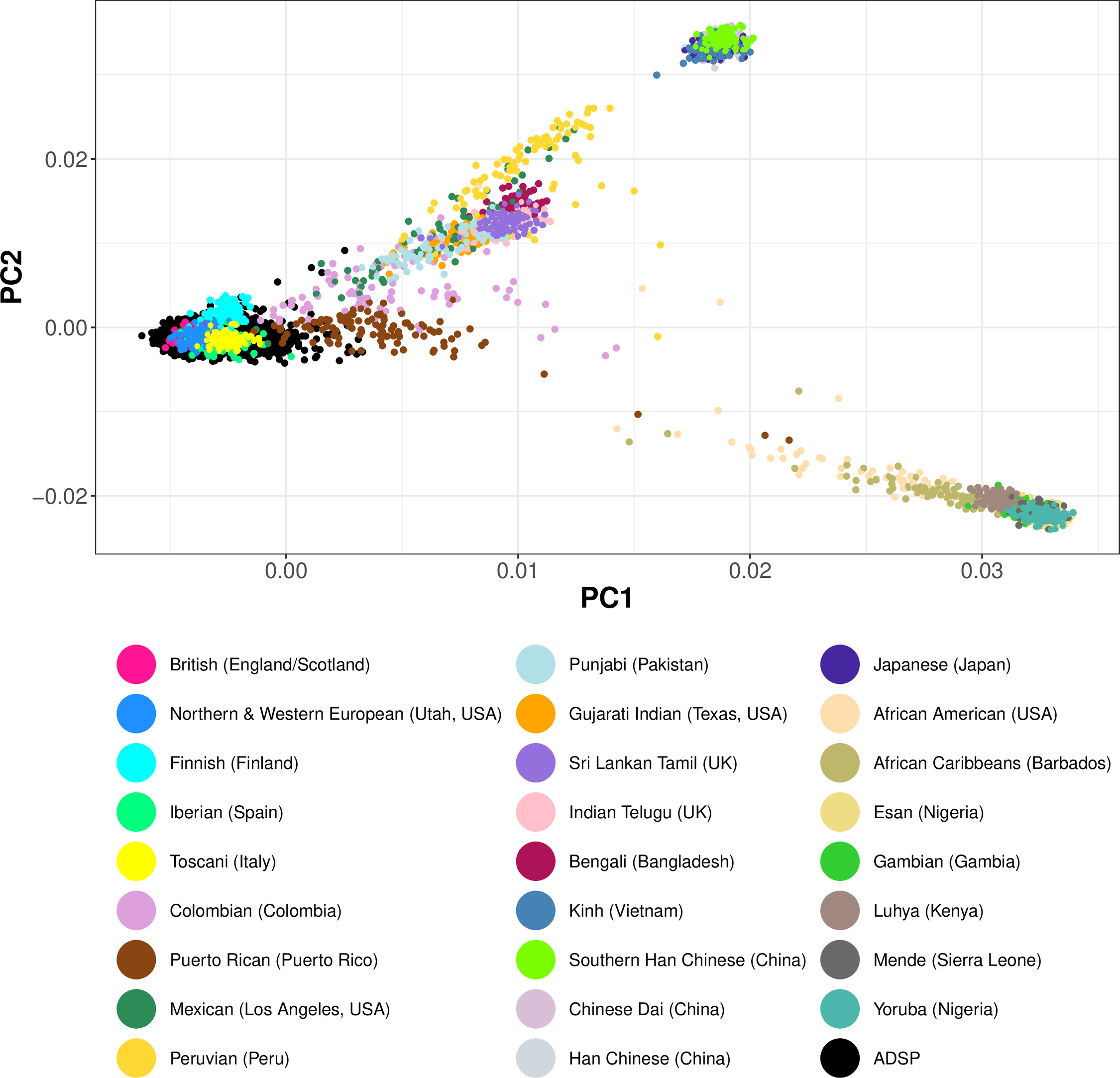


**S1 Fig. Principal Component (PC) eigenvector plot of combined 1000 Genomes and ADSP genotypes.**

Supplement: S1 Fig — Each data point represents a single individual. 1000 Genomes reference individuals are color-coded by ancestry. ADSP samples are shown in black. The position of ADSP samples relative to the 1000 Genomes reference samples indicates their genotypic similarity, which reflects ancestry. Most ADSP samples cluster near European reference samples (e.g. Finland and Spain). (DOCX) [file pone.0249305.s001.docx]
