# Supplementary material for: Impact of variant-level batch effects on identification of genetic risk factors in large sequencing studies
Source: PLoS One. 2021 Apr 16;16(4):e0249305. doi: 10.1371/journal.pone.0249305 (PMC8051815; doi:10.1371/journal.pone.0249305)
Supplement: S2 Fig — These variants totaled 120,572 from the Center 1 samples; 108,390 from the Center 2 samples; and 98,542 from the Center 3 samples. Approximately 70% of variants were shared among samples from all three sequencing centers. The larger number of variants detected in Center 3 samples was likely due to the larger number of individuals sequenced by Center 1 compared to the other two centers. (DOCX) [file pone.0249305.s002.docx]

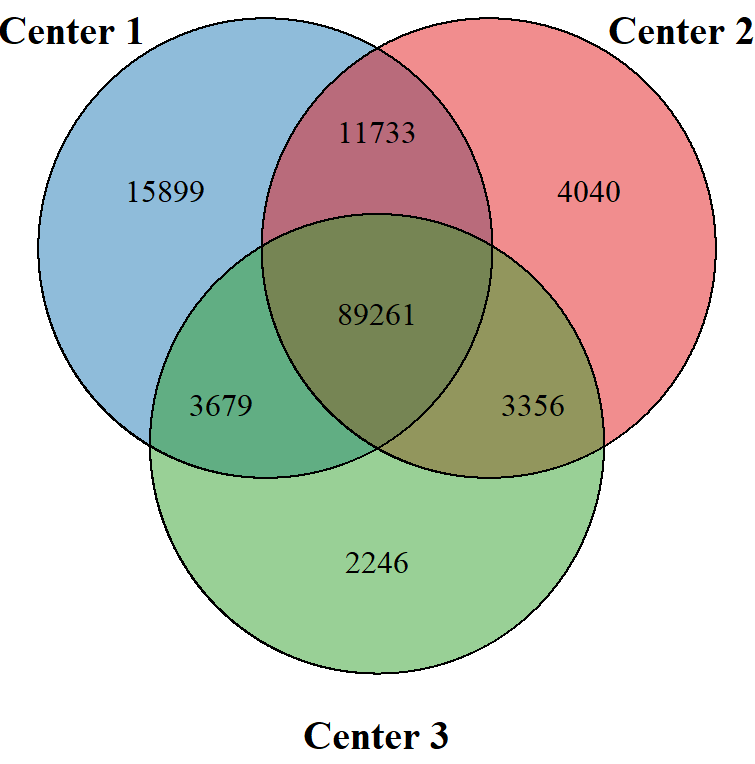


**S2 Fig. Number of QCed variants used for association analysis, and their overlap among samples from three sequencing centers.**
