# Supplementary material for: Impact of variant-level batch effects on identification of genetic risk factors in large sequencing studies
Source: PLoS One. 2021 Apr 16;16(4):e0249305. doi: 10.1371/journal.pone.0249305 (PMC8051815; doi:10.1371/journal.pone.0249305)
Supplement: S3 Fig — The red vertical line denotes exome-wide statistical significance (p < 3.0 x 10^−7^). (DOCX) [file pone.0249305.s003.docx]

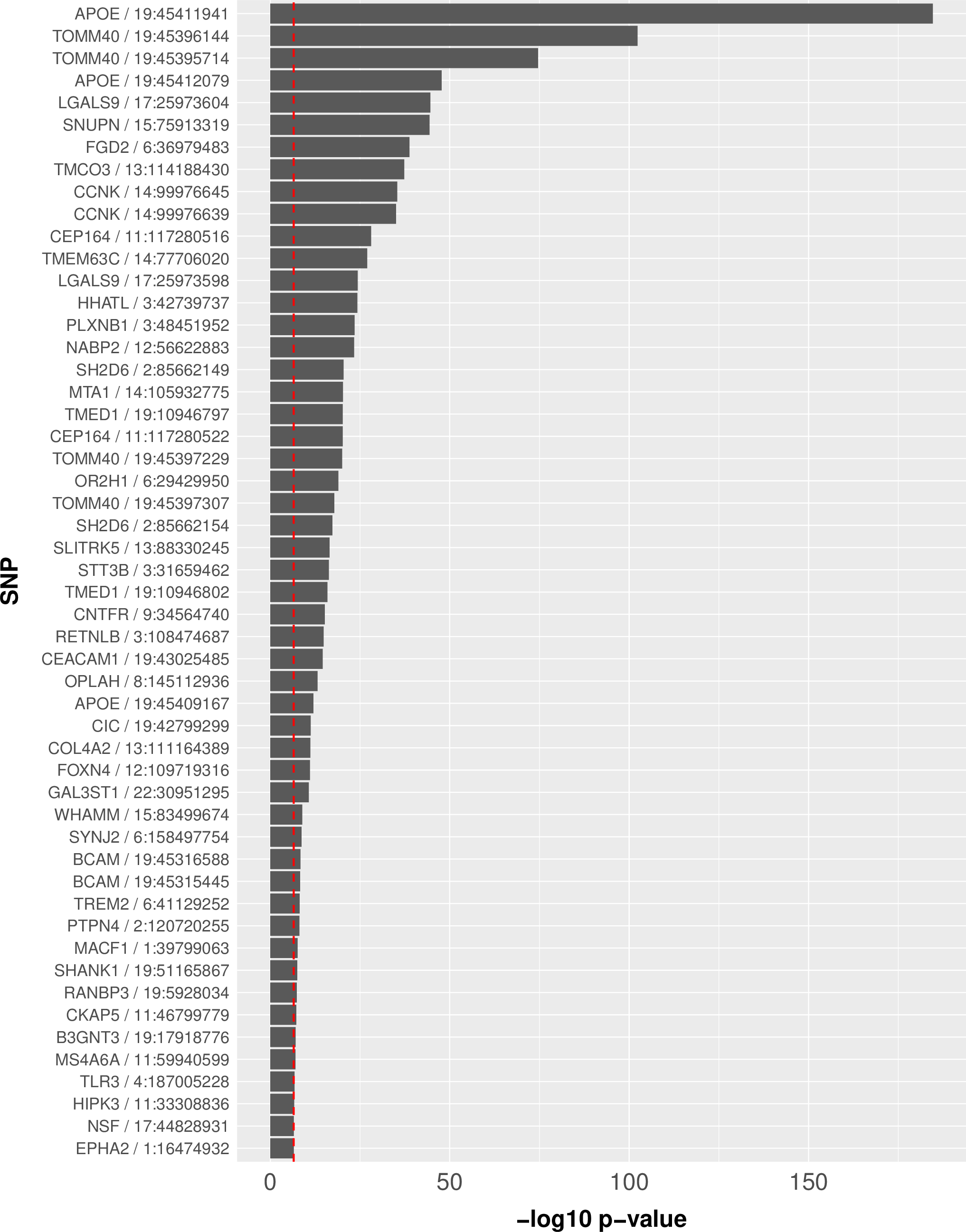


**S3 Fig. Log-transformed p-values of all 52 SNPs reaching exome-wide significance under any association model (largest p-value shown for each SNP).**
