# Supplementary material for: Impact of variant-level batch effects on identification of genetic risk factors in large sequencing studies
Source: PLoS One. 2021 Apr 16;16(4):e0249305. doi: 10.1371/journal.pone.0249305 (PMC8051815; doi:10.1371/journal.pone.0249305)
Supplement: S4 Fig — Mean values were computed across all samples for each variant. Solid vertical lines demarcate the boundaries separating the 5% tails from the middle 90% of the distribution. The scattered dots represent the positions of the top 29 SNPs within the distributions. Almost all top SNPs lie far to the right of the mean AAF ratio distribution, indicating that these variants are highly discrepant between capture kits. (DOCX) [file pone.0249305.s004.docx]

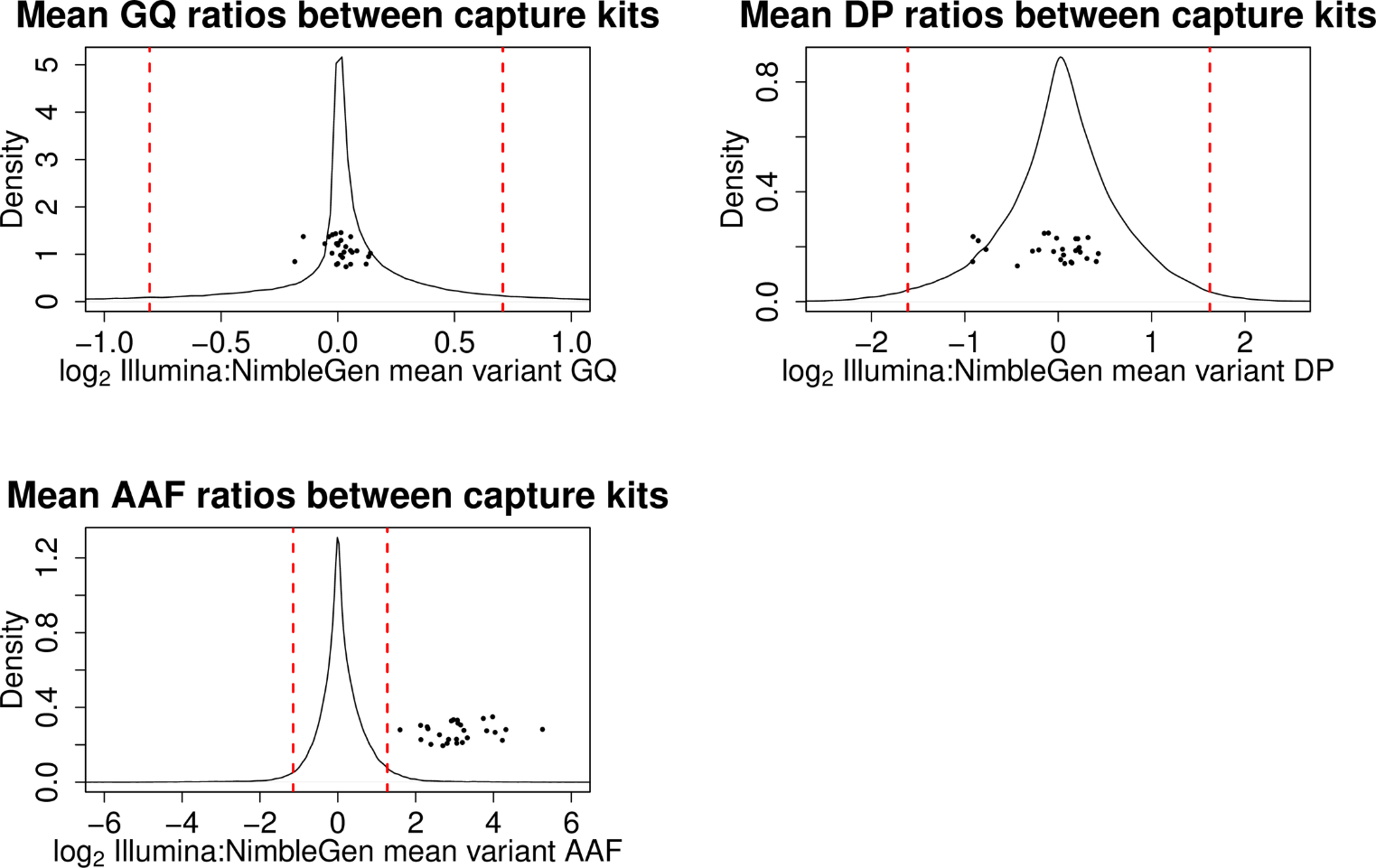


**S4 Fig. Distributions of log-adjusted ratios of mean quality metrics for QCed variants shared between capture kits.**
